# Spontaneous Expression of Neurotrophic Factors and TH, Nurr1, Nestin Genes in Long-term Culture of Bone Marrow Mesenchymal Stem Cells

**Published:** 2011-12-22

**Authors:** Fatemeh Moradi, Maryam Haji Ghasem Kashani, Mohammad Taghi Ghorbanian, Taghi Lashkarbolouki

**Affiliations:** Department of Biology, Damghan University, Damghan, Iran

**Keywords:** Bone Marrow, Mesenchymal Stem Cells, long-term culture, Spontaneous Differentiation, Neurotrophic Factors

## Abstract

**Objective:**

It has been reported that rat bone marrow stromal cells (BMSCs) can be spontaneously differentiated into neural-like cells without any supplemental growth factors and/or chemical treatment after long-term culture.This study aims to determineWhether, growth factors secreted by MSCs could induce self-differentiation into neural-like cells in a long-term culture.

**Materials and Methods:**

This study consisted of two groups: i. rat BMSCs (passage 5) were cultured in alfa- minimal essential medium (α-MEM) and 10% fetal bovine serum (FBS) without the addition of inducer and exchanging medium for three weeks, as the experimental group and ii.rat BMSCs (passage 5) as the control group. Each group was analysed by reverse transcriptase polymerase chain reaction (RT-PCR) to evaluate the expressions of neurotrophic factors and neural marker genes.

Statistical analyses were carried out using one-way analysis of variance (ANOVA) and Tukey’s multiple comparison with SPSS software (version 16). P< 0.05 was considered statistically significant.

**Results:**

The experimental group (fifth passage of BMSCs) obtained from adult rats spontaneously differentiated into neural precursor cells after long-term culture. Cultured cells expressed tyrosine hydroxylase (TH), Nurr1 and nestin genes. Furthermore, some growing cells in suspension became neurosphere-like. Self-differentiated rat MSCs (SDrMSCs) expressed significantly higher levels of NGF (0.96 ± 0.16), nestin (0.63 ± 0.08), and Nurr1 (0.80 ± 0.10) genes (p<0.05).

**Conclusion:**

In this study, we reported that rMSCs in long-term culture underwent spontaneous transformation to neural precursors without the supplement of growth factors and specific chemicals. Cells expressed neural markers such as: TH, Nurr1, and nestin genes.

## introduction

Bone marrow contains at least two populations of multipotent cells, hematopoietic stem cells (HSCs) and non-hematopoietic stem cells. Nonhematopoietic stem cells are also called mesenchymal stem cells (MSCs), bone marrow stromal cells (BMSCs), or colony-forming-unit fibroblasts. These cells reside in the bone marrow stromal system (1, 2). BMSCs were discovered by Friedenstein (3, 4)who has described them as clonal, plastic adherent cells that were able to differentiate into osteoblasts, adipocytes, and chondrocytes (3, 5). These cells are also stromal cells, or structural components of the bone marrow that support ex vivo culture of haematopoiesis by providing extracellular matrix components, cytokines and growth factors (3, 6).

BMSCs are of interest due to their possible use as cell therapy in neurological diseases. These cells are multipotent and easily available from aspirates of whole bone marrow andcan be isolated because they adhere to the tissue culture surface (1, 7).

Rat BMSCs may also differentiate in vitro into cells of non-mesodermal origin, such as neurons, skin and gut epithelial cells, hepatocytes and pneumocytes (5). Several *in vitro* studies have described conditions under which BMSCs can be differentiated into neural-like cells. These conditions included chemical inducers, cytokines, chemical inducers plus cytokines, special supplements plus cytokines, and co-culturing with neurons or glia (8, 9).

In a recent study,non-induced, serum-free rat BMSCs expressed neural marker genes without any induction (10).Expressions of several neural genes, including neurogenic transcription factor neuroD, nestin, NeuN, microtubule-associated protein-2 (MAP-2), tyrosine hydroxylase (TH), and glial fibrillary acidic protein (GFAP) by marrow stromal cells, even before induction has been confirmed and indicated by several studies (11-14).

In a recent investigation, mouse BMSCs spontaneously expressed certain neuronal phenotype markers in culture, in the absence of specialized induction reagents (15). Li and co-workers have reported spontaneous expression of nerve growth factor (NGF), TrkA, and TrkB genes in a long-term culture (16).

The mechanism for transdifferentiation of BMSCs is unclear, but may result from induction of neurotrophic factors (NTFs) (16, 17). NTFs are a family of growth factors that consist of NGF, brain-derived neurotropic factor (BDNF), neurotrophin- 3 (NT-3), and neurotrophin-4/5 (NT-4/5) in mammals. They are critical for neural survival, development, functional maintenance and plasticity of the central nervous system (CNS) (18).

Cultured BMSCs in DMEM medium secrete NGF, BDNF, GDNF, and NT-3 (1). BMSCs express several neurotrophic factor genes including NGF, BDNF, ciliary neurotropic factor (CNTF), and insulin-like growth factor-1 (IGF-1), which promote survival of neuroblast cells and neurogenesis in vitro (1, 19, 20), thus indicating their therapeutic role in the protection of the injured central nervous system.

This study aims to determine if rat BMSCs could be differentiated spontaneously into neural precursor cells and express neural markers genes in the absence of specialized induction reagents by secreting neurotrophic factors in a long-term culture.

## Materials and Methods

### Rat MSCs culture

Adult Sprague-Dawley rats (4-6 weeks old ) were purchased from Razi Institute, Karaj, Iran and kept at standard conditions, according to the guidelines of Damghan University Animal Ethics Committee for minimal animal discomfort. Briefly, animals were sacrificed, then their tibias and femurs were removed. BMSC culture media (5 ml) that consisted of α-MEM (Invitrogen Gibco-USA; cat. 11900-073) supplemented with 10% fetal bovine serum (FBS; Gibco, USA) and1% penicillin/streptomycin was injected into the central canal of the bones to extrude the marrow. Whole marrow cells were extracted and cultured in 25 cm^2^ culture flasks at a density of 5-10×10^5^ cells/cm^2^ and incubated at 37℃ with 5% humidified CO_2_. Non-adherent cells were removed after 72 hours by changing the media. The medium was replaced every 2-3 days. Confluent cells were split at a ratio of 1:2 by using 0.25% trypsin and 0.02% EDTA, then passaged five times. Control samples were collected from this passage. Sub-confluent rat BMSCs (passage 5) were cultured in the same media for three weeks. During this time, the media was not changed nor supplemented with additional factors.

### Immunocytochemistry

Identification of the different cell types was performed by immunocytochemistry. BMSCs (passage 5) were identified by using Millipore's Alkaline Phosphatase Detection Kit (Catalog number SCR004, USA) (21, 22) and primary antibody that included monoclonal anti- human CD71 (Sigma; C2063) and fluorescein isothiocyanate (FITC) labelled antibody to CD71 as the secondary antibody . Long-term cultured MSCs were identified by primary antibody that included rabbit anti-nestin (Sigma; N5413) and FITC-labelled antibody to nestin as the secondary antibody (Chemicon; AP 132F).

Cells were fixed in 4% paraformaldehyde at room temperature for 20 minutes and washed three times with PBS for 5 minutes each time. Cells were then treated with 0.3% Triton X-100 that contained 10% normal goat serum at room temperature for 30 minutes. Cells were incubated with primary antibody at 4ºC for 18-24 hours. After washing three times with PBS for 5 minutes, FITC-conjugated secondary antibody was added to the cells. Cells were then incubated at 37ºC for 1 hour followed by two more rinses in PBS for 5 minutes each. The slides were examined by florescence microscopy.

### Reverse transcriptase polymerase chain reaction

Cultured cells were observed daily, and experimental samples were collected on day 21. Control and experimental samples were analysed by RTPCR. Total RNA was extracted from both control (passage 5) and long-term culture rat BMSCs (3 weeks) by using RNX-Plus solution (1 ml/10^6^ cells; CinnaGen).The resultant RNA pellet was subjected to a chloroform extraction and two ethanol precipitations. RNA concentrations were determined by measuring the absorbance at 260 nm (Eppendorf, Germany).

Table 1 displays the primer probe sets used for the RT-PCR experiments. β2M was used as the house-keeping gene. The standard reverse-transcription reaction was performed with 0.5 µg total RNA using oligo(dt) as a primer and the Revert Aid H Minus First Strand cDNA Synthesis Kit (Fermentas-k1622) according to the manufacturer’s instructions. Subsequent PCR was as follows: 3 µl cDNA, 1x PCR buffer, 200 µM dNTP, 0.5 of each primer pair, and 0.25 unit/25 µl reaction Taq DNA polymerase. PCR was carried out in a master cycler. PCR reaction conditions were as follows: one cycle: 94℃, 2 minutes; 94℃, 30 seconds; 55℃, 30 seconds; 72℃, 30 seconds; 3 rep (34), 72℃, 5 minutes. After RT-PCR, the DNA products were electrophoresed on 1.5% agarose gel that contained ethidium bromide. Gene bands were observed under ultra violete (UV) light and photographed.

### Statistical analyses

Statistical analyses were carried out by using one-way ANOVA with Tukey’s multiple comparison. For each parameter, the significance level was determined by using SPSS (Version 16).

## Results

### Rat MSCs characterization

**Fig 1:**
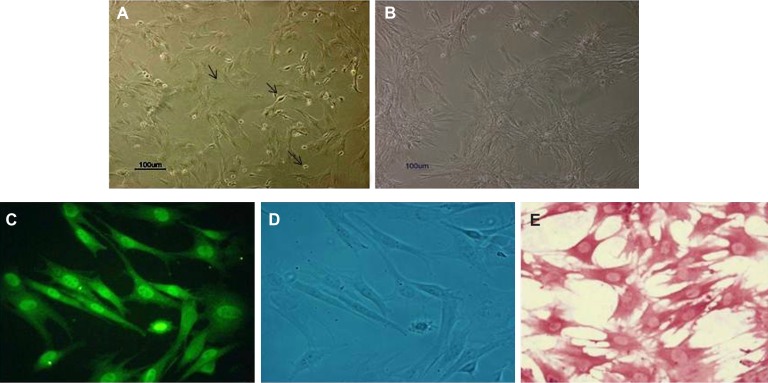
Characterization of MSCs culture. A. During the onset of culture, BMSCs showed morphologies that were round, bipolar, large and flattened. B. After five passages, BMSCs exhibited fibroblast-like morphology and became flattened. Scale bar = 100µm. Most BMSCs were immunoreactive to CD71 .C. Phase-contrast photomicrograph. D. Alkaline phosphatase E. (× 400)

Rat BMSCs isolated from bone marrow suspensions by selective attachment to plastic tissue culture flasksexhibited a heterogeneous appearance, which included round, bipolar or large flat shapes (Fig 1A). However, after reaching confluence, rat BMSCs became morphologically homogeneous with two types of cells, small fibroblastlike and large flattened morphologies (Fig 1B).

**Table 1:** The primers used for reverse transcription-polymerase chain reaction (**RT-PCR**) analysis


Gene	Predicted size (base pairs)	Primer sequence	Accession number
NGF	164 bp	F: 5'-CCT-CTT-CGG-ACA-CTC-TGG-<A>-3' R: 5'-CGT-GGC-TGT-GGT-CTT-ATC-<T>-3'	NM_012610
BDNF	405 bp	F: 5'-GCC CAA CGA AGA AAA CCA TA-3' R: 5'-GAT TGG GTA GTT CGG CAT TG-3'	D 10938
NT3	181 bp	F: 5' -AGG TCA GAA TTC CAG CCG AT-3' R: 5' -GTT TCC TCC GTG GTG ATG TT-3'	NM_031073
NT4/5	213 bp	F: 5'-TAT GTG CGG CGT TGA CTG C-3' R: 5'-CAC AGT CAG AAG GCA CGG TA-3'	NM_ 013184
GDNF	254 bp	F: 5'-GAC TCC AAT ATG CCC GAA GA-3' R: 5'-TAG CCC AAA CCC AAG TCA GT-3'	NM_019139
β2M	318 bp	F: 5'-CCG TGA TCT TTC TGG TGC TT-3' R: 5'-TTT TGG GCT TCA GAG TG-3'	NM_012512
Nurr1	683 bp	F: 5'-TCC CGG AGG AAC TGC ACT TCG-3' R: 5'-GTG TCT TCC TCT GCT CGA TCA-3'	U72345
TH	276 bp	F: 5'-TGT CAC GTC CCC AAG GTT CAT -3' R: 5'-CGT GGG ACC AAT GTC TTC AGT G- 3'	NM_012740
Nestin	431 bp	F: 5'- CAG- GCT –TCT- CTT- GGC- TTT- CTG- <G>-3' R: 5'- TGG- TGA- GGG -TTG -AGG -TTT-G- <T>- 3'	NM_012987


**Fig 2:**
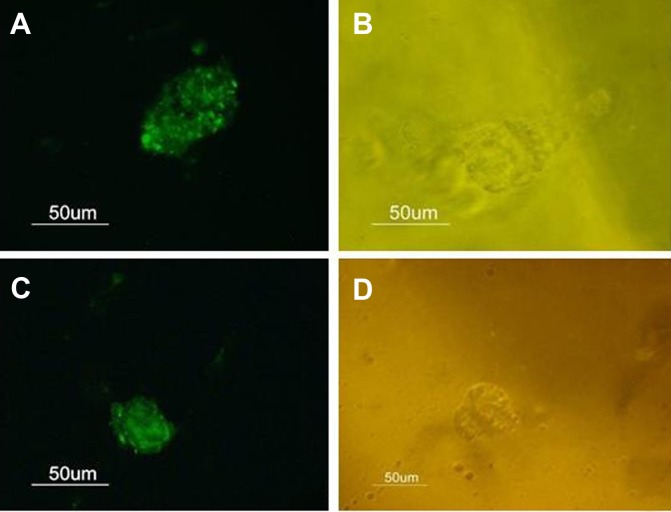
A, C: Nestin-immunoreactive cells could be detected in a subset of the BMSCs after longterm culture. B, D: Phase-contrast photomicrograph of nestin-immunoreactive cells. Scale bar = 50 µm

Both types of cells expressed CD71 (Figs 1C, D) (23, 24) and reacted to alkaline phosphatase (Fig 1E). More than 95% of the cells were immunopositive. However, rat BMSCs began forming floating cell masses and transformed to nestin-positive neurospheres (a neural stem cell marker), after three weeks of culture (Figs 2A, C). Phase contrast photomicrographs of the same fields are observed in figures 2B, D.

### Characterization of MSCs after long-term cultivation

**Fig 3:**
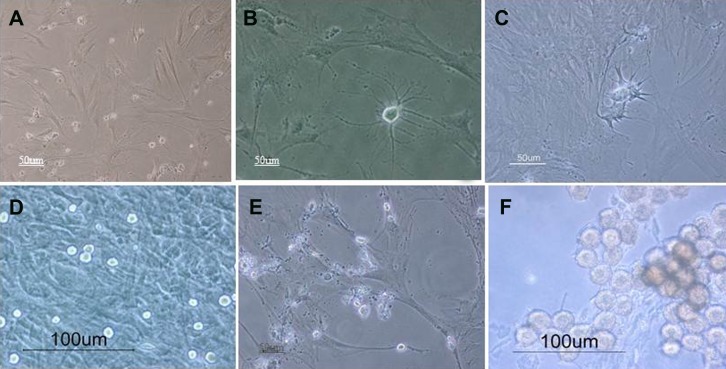
Phase contrast image of P5 rat BMSCs, which were cultivated for three weeks without passage after the first week (A);second week (B,C); and third week. These cells formed multiple cellular clumps and showed neurosphere-like appearance (D, E, F). Scale bar = 50 µm (A,B, D-F) , 20 µm (C)

MSCs were cultured in unchanged media for at least three weeks. After the first week, the cells were spindle-like and able to proliferate (Fig 3A). During the second week, the cells began to change into neural-like cells and developed long, thin projections (Fig 3B, C).

As they were growing, parts of the cells would detach from the wall; these cells formed multiple cellular clumps that showed a neurosphere-like appearance. (Fig 3D, E, F).

**Fig 4:**
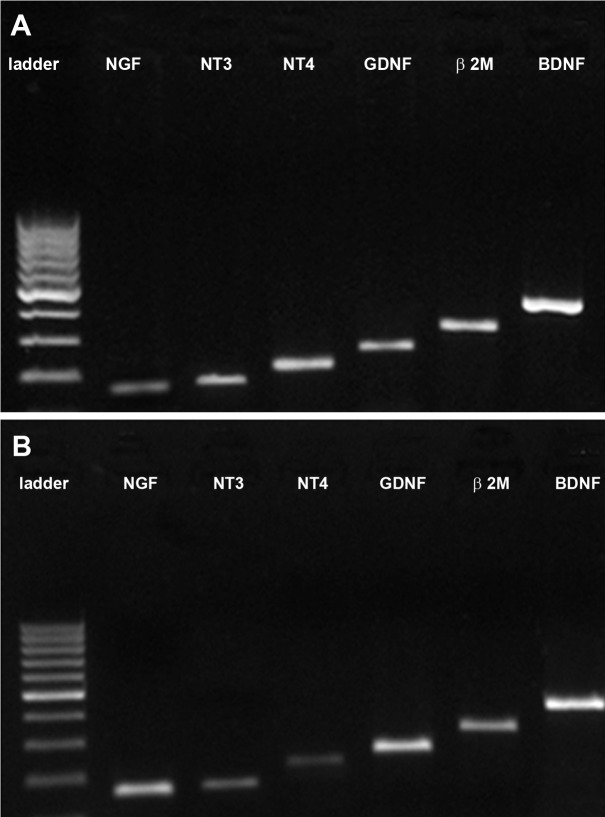
Detection of of NGF, NT3, NT4/5 band, GDNF and BDNF mRNA from rat bone marrow stromal cells (BMSCs) by RT-PCR. A: 164- bp NGF band, 181- bp NT3 band, 213- bp NT4/5 band, 254- bp GDNF band and 405- bp BDNF band were detected in BMSCs after five passages (control) and B: long-term culture (3 weeks). β2M is a housekeeping gene (318 bp).

**Fig 5:**
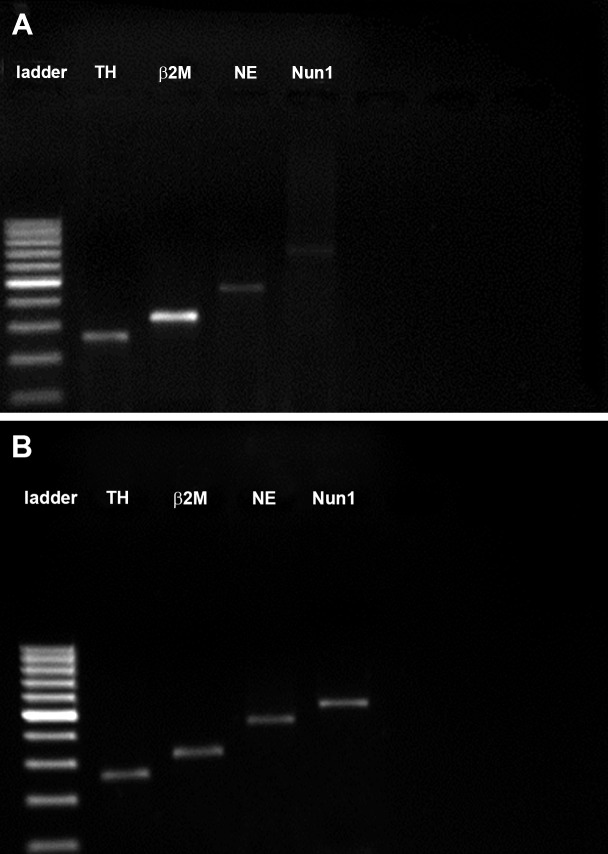
Detection of of TH, NE, Nurr1 and mRNA from rat bone marrow stromal cells by RT-PCR. A: 272-bp TH band, 318-bp β2M band (housekeeping gen), 431-bp nestin and 683-bp Nurr1 band were detected in BMSCs after five passages (control), and B: long-term culture (three weeks).

### **RT-PCR** analysis

**Fig 6:**
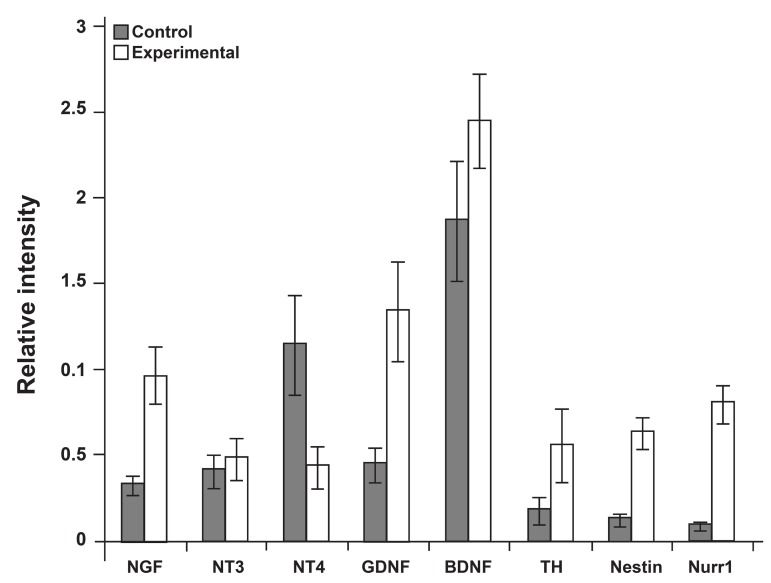
Band intensity of **RT-PCR** results in fifth passage (control) and three week cultured (experimental) MSCs. NGF (0.96 ± 0.16), Nestin (0.63 ± 0.08) and Nurr1 (0.8 ± 0.10) were significantly increased in the exprimental group compared with the control group. Bars show means ± SE values. ^*^p <0.05.

RT-PCR analysis confirmed the gene expressions of neurotrophic factors such as BDNF, NGF, NT-3, NT-4/5, GDNF (Figs 4A, B) and neural markers such as Nurr1, TH and nestin in rat BMSCs that were cultured for 3 weeks (Figs 5 A,B). These results were compared with the control group. As shown in figure 6, the level of NGF increased significantly in the experimental (0.96 ± 0.16) group compared with the control (0.32 ± 0.05) group (p<0.05). All neuronal marker genes were detected in both groups, but nestin and Nurr1 levels for SDrMSCs at 3 weeks were 0.63 ± 0.08 for nestin and 0.80 ± 0.10 for Nurr1compared to 0.12 ± 0.03 (nestin) and 0.09 ± 0.02 (Nurr1) in the control group of rat BMSCs (p<0.05).

## Discussion

In this study, rat BMSCs were evaluated for CD71 and alkaline phosphatase expression. The results showed that, the fifth passage of these cells were 95% positive for CD71 and alkaline phosphatase, which had been used to characterize BMSCs.

In this work, we showed that MSCs derived from bone marrow were able to express neural markers without external induction.

When the MSCs grew to confluency, they lost contact inhibition and proliferated continuously. Under long-term cultivation (3 weeks) without passage, they formed cellular aggregations with rosette-like appearances. Rosette-like cellular structure was also observed when the embryonic cells differentiated into neural precursor cells (23).

In a recent report it was demonstrated that primary MSCs obtained from adult rats could spontaneously differentiate into neural precursor cells after 6 weeks (25). It was reported that adipose-derived stem cells (ADSCs) differentiated spontaneously into immature neural-like cells after *in vitro* longterm culture (26). In addition, prolonged cultured human embryonic stem cells expressed neural precursor markers such as nestin and neural cell adhesion molecule (NCAM). These cells could be induced into neurons by serum deprivation and retinoic acid supplementation (27).

BMSCs had been used as nutrient-providing cells to support the growth and differentiation of neural stem cells by providing growth factors (19, 28, 29). Many growth factors have key roles in the differentiation of rat BMSCs into neural cells. BMSCs have been reported to express neurotrophins and their high affinity receptors (16) that were important in the development, regeneration and survival of neural cells (30). In a recent study, the expression of BDNF, GDNF, NGF, NT3, and NT4/5 genes was also determined in non-treated BMSCs (31).

In a recent study, it was shown that BMSCs secrete NTFs and facilitated neural repair (11,30, 32), recruited supporting cells, or restored injured tissues (33). After being grafted into the injured central nervous system, neural cells derived from BMSCs settled at the injury site directly, replaced lost neurons, and secreted growth factors that could provide a micro-environment to promote grafted cell survival (31, 34).

Thus, we investigated whether growth factors secreted by MSCs could induce spontaneous differentiation into neural-like cells. In some cases, the cells even became neurospheric.

A previous study has shown that long-term cultivation of adult rat BMSCs spontaneously enriched nestin-positive neural precursors. These neural precursors derived from long-term cultures were more sensitive to neural induction by serum deprivation and growth factor supplementation than cells obtained from normal sub-confluent cultures (25). In addition, nestin expression by these cells and their ability to grow in suspension in culture conditions brought them nearer to a neurosphere phenotype. These changes may support that rat BMSCs can differentiate into neural-like cells, since it has been reported that non-neural stem cells become neurospheric before trans-differentiation into neural cells (35, 36).

We examined mRNA levels of BDNF, NGF, NT-3, NT-4/5, GDNF, TH, Nurr1 and nestin in rat BMSCs after 3 weeks by RT- PCR. Significant increases of nestin and Nurr1 genetic expressions indicated that rat BMSCs in long-term culture underwent spontaneous transformation to neurallike cells and were able to express neural markers without cytokines or specific chemicals. Secretion of neurotrophic factors induced expression of neuronal genes such as nestin and Nurr1 in the experimental group. It was concluded that BMSCs have the potential for self-differentiation into dopaminergic neurons in long-term culture, but by electrophysiological analysis we can determine that these cells are really functional neurons.

Further studies on self-differentiation are necessary to determine: i. whether SDrMSCs are functional neural cells; ii. if actual functional specifications are achieved, rMSCs could become an appropriate cellular model for the study of neurological diseases; and iii. it is important to elucidate which factors can protect against neuronal cell death in specific diseases and to understand which cellular pathways activated by NTs are responsible for their protective effects in an in vivo brain damage model, such as cerebral ischemia or brain trauma.

## Conclusion

From our experiments, we have proven that rat BMSCs could convert into neural phenotype and express neural markers such as TH, Nurr1, and nestin in long-term culture. This transformation was natural and even spontaneous, without induction by cytokines or specific chemicals.

It was suggested that BMSC-derived neurospheres could be differentiated into dopaminergic neurons in the appropriate culture conditions and growth factor supplements. Thus, these cells will be an important source of cells for cell therapy in patients with Parkinson’s disease.
